# Degradation Measurement and Modelling under Ageing in a 16 nm FinFET FPGA

**DOI:** 10.3390/mi15010019

**Published:** 2023-12-22

**Authors:** Justin Sobas, François Marc

**Affiliations:** IMS laboratory, University of Bordeaux, CNRS UMR 5218, Bordeaux INP, F-33400 Talence, France; francois.marc@u-bordeaux.fr

**Keywords:** FPGA, FinFET, ageing, BTI, HCI, ring oscillator

## Abstract

Most of the latest generation of integrated circuits use FinFET transistors for their performance, but what about their reliability? Does the architectural evolution from planar MOSFET to FinFET transistor have any effect on the integrated circuit reliability? In this article, we present a test bench we have developed to age and measure the degradation of 5103 ring oscillators (ROs) implemented in nine FPGAs with 16nm FinFET under different temperature and voltage conditions ( $V_{nom}\leq V_{stress}\leq 1.3V_{nom}$ and $25\;{}^{\circ}C\leq T_{stress}\leq 115\;{}^{\circ}C$ ) close to operational conditions in order to predict reliability regarding degradation mechanisms at the transistor scale (BTI, HCI and TDDB) as realistically as possible. By comparing our initial RO measurements and the data extracted from Vivado, we will show that the performance of the nine FPGAs is between 50% and 70% of the best performance expected by Vivado. After 8000 h of ageing, we will see that the relative degradations of the RO are a maximum of 1% , which is a first indicator proving the FPGAs’ good reliability. By comparing our results with similar studies on 28 nm MOSFET FPGAs, we will reveal that 16 nm FinFET FPGAs are more reliable. To be implemented in an FPGA, an RO uses logic resources (LUT) and routing resources. We will show that degradation in the two types of resources is different. For this reason, we will present a method for separating degradations in logical and routing resources based on RO degradation measures. Finally, we will model rising and falling edge propagation time degradations in an FPGA as a function of time, temperature, voltage, signal duty cycle and resources used in the FPGA.

## 1. Introduction

### 1.1. Context

In the 2010s, developments in planar transistor (MOSFET) architecture pushed back the limits of scaling and led to the emergence of the FinFET. The FinFET, a transistor with an out-of-plane fin-shaped channel, is widely used in the latest generation of digital circuits with nodes down to five nanometers [1]. The reliability of a digital circuit under ageing effects is affected by degradation at the transistor level [2] (chapter 15) but also by defects at the packaging level [2] (chapter 16). This article will focus on transistor degradation without considering the other mechanisms. The reliability of a transistor under the ageing effect is affected by three main degradation mechanisms: Bias Temperature Instability (BTI) [3], Hot Carrier Injection (HCI) [4] and Time-Dependent Dielectric Breakdown (TDDB) [5]. The common failure mode for BTI and HCI is the progressive degradation of the transistor threshold voltage, and the failure mode for TDDB is the leakage current in the gate oxide. In a digital circuit, these failure mechanisms induce an increase in signal propagation time [6] (Table 1). While the three degradation mechanisms are widely observed and modelled in planar transistors, with more than 5600 publications for BTI between 2006–2016 [7], the following question emerges: What about reliability under the ageing effect of FinFET transistors and its impact on digital circuits?

### 1.2. State-of-the-Art FinFET Ageing

The state of the art in FinFET reliability analysis already gives us an idea of the answer to the previous question. By comparing the degradation of a 16 nm FinFET with a 28 nm HKMG MOSFET, TSMC [8] shows that PBTI decreases while NBTI remains unchanged. Intel also shows in [9] that PBTI decreases in the 22 nm FinFET compared with the 32 nm HKMG MOSFET; however, a slight increase in NBTI is observed in the FinFET. In [10], the author reveals that the NBTI recovery mechanism is more important in a 20 nm MOSFET than in a 14 nm FinFET, which may explain why the overall degradation due to NBTI is greater in the FinFET [9]. A simulation based on the diffusion–reaction model of a 16 nm MOSFET and FinFET is performed in [11] and indicates that the propagation delay degradation is 26% higher in MOSFET. In [9], the author shows that the HCI is lower in the FinFET than in the MOSFET for low drain voltages, and this trend reverses for high drain voltages. This study also shows that the HCI is higher when the width of the channel decreases due to the SHE (Self-Heating Effect). The SHE is also the reason why the HCI is higher as the number of fins increases [12].

### 1.3. Are Predictions of Transistor Reliability under Ageing Based on a Few Hours of Measurements Realistic?

To develop a physical model of degradation, such as the reaction–diffusion model for BTI [13] or the lucky electron model for HCI [14], measurements need to be made at the transistor level. However, such access requires the following [15]:

- A test board dedicated to measuring transistor degradation;
- Probes, sometimes nanometric, depending on the dimensions of the transistor being measured;
- Specific instrumentation to place the probes and measure the threshold voltage.

It is expensive to monopolise such a test bench with access to the transistor, so most of the degradation measurements are carried out for less than 10,000 s (≈3 h) [10,12,16,17]. In order to observe degradation over a short test period, the ageing conditions applied are far from operational conditions. In [18,19], a stress voltage almost three times higher than the nominal voltage is applied. For BTI modelling, the stress temperature of the transistor is generally 125 °C [18,20,21,22]. Under these extreme conditions and for just a few hours of measurement, the average degradation measured is between 3–8% and corresponds to the degradation predicted for three years of use under operational conditions, which represents an acceleration factor of around 2000 [17]. Applying such high stresses can result in the occurrence of failure mechanisms which are not representative of those present under operational conditions. Degradation measurements at the transistor level are necessary to develop physical models but more uncertain for predicting the reliability of a component or circuit over several years under operational conditions. This section highlights the need for a new method, other than the expensive transistor-level measurement, to perform long-term ageing tests under moderate conditions.

### 1.4. Measuring Degradation in an FPGA

The common failure mode of the BTI, HCI and TDDB, the degradation of the threshold voltage of the transistor, increases the propagation time of a signal in a digital circuit. For more than 10 years, methods have been developed such as the transition probability [23] and the ring oscillator [24] to measure the degradation of signal propagation time in an FPGA. Here is a review of the methods for measuring degradation in an FPGA [25]. A low-cost test bench for measuring degradations in FPGAs is presented in [24]. Because the test bench is low-cost and the measurements are automatic, a 12,000 h ageing test was performed [26] and revealed final degradations of around 2% for a stress voltage that is only 30% higher than the nominal voltage. Semiempirical models of BTI [27] and HCI [27] are developed from the measurements. The comparison of the conditions and results of ageing tests on FPGAs that we have conducted in [28] (Table 1), based on ten studies, highlights the importance of measurement accuracy so as to observe degradations under stress conditions close to operational conditions. We developed a test bench [28] for measuring degradations in an FPGA with a relative accuracy of 0.009% , which classifies it as the most accurate in the state of the art.

### 1.5. Purpose of the Article and Plan

In this article, we present the results of an ageing test of 8000 h on nine FPGAs based on 16 nm FinFET. The new features of the article are as follows:

- Before ageing, we compare the propagation times measured with those estimated by the design software (Vivado ML 2023.2);
- After 8000 h of ageing, we present the degradations measured in 5103 ring oscillators split between nine FPGAs with temperatures between 25 °C and 115 °C and voltages from $V_{nom}$ to 1.3 $V_{nom}$ ;
- We compare our degradation results with studies carried out on 28 nm MOSFET FPGAs to relate the evolution of reliability from MOSFET to FinFET;
- We present a new method to separate degradations in logic and routing resources in the FPGA;
- We propose a semiempirical model to predict degradation as a function of temperature, voltage, cyclic ratio and the resources used in the FPGA.

We describe the test bench in Section 2. In Section 3, we present the results that we measured on the 5103 ROs before and after 8000 h of ageing, and we also compare our degradations measured on a 16 nm FinFET FPGA with those measured on a 28 nm HKMG FPGA. In order to model degradations in FPGA logic and routing resources separately, in Section 4, we introduce a method we developed for extracting degradations in both resources. Finally, we model the propagation time degradations and compare them with the critical limit set by Vivado in Section 5.

## 2. Presentation of the Test Bench

### 2.1. Methodology

The objective of the test bench is to age and measure propagation time degradation in an FPGA. The general method consists in implementing the ring oscillator (RO) in the FPGA and measuring its oscillation frequency.

A ring oscillator is a circuit composed of *n* stages whose output is looped back to the input, and the signal must be inverted each time it passes through the circuit. Thus, the output signal of an RO oscillates with a period:

(1) $$T_{RO}=2\times \sum_{i=1}^{n}\tau_{i}$$

where $\tau_{i}$ is the signal propagation time in the *i*th stage of the RO and *n* is the number of stages in the RO.

**Figure 1:**
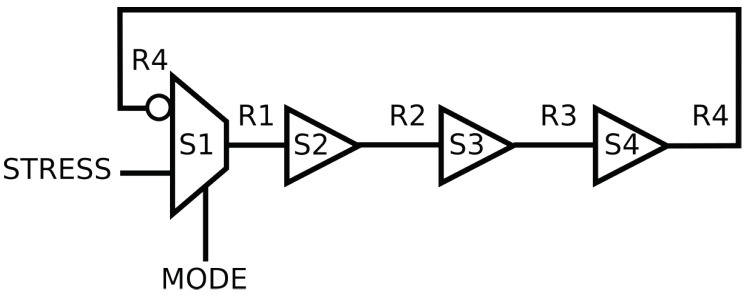
Schematic of four stages of RO with a multiplexer to control the input signal and buffers.

To control the operating mode of the RO, we implement a multiplexer as the first stage of the RO (see Figure 1). In measurement mode, the RO loop is closed in order to measure the RO signal. In stress mode, which corresponds to 99.7% of the time, the RO loop is open and a stress signal passes through the RO. This enables us to study the effect of the stress signal (internal stress) on RO degradation.

The measurement circuit architecture is based on that developed in [24]. The circuit measures the frequency and duty cycle of the ROs using counters implemented in the FPGA. From the oscillation frequency measurement of the RO, we know its period. To further our analysis, from the RO oscillation period and its duty cycle ( $\alpha_{RO}$ ), only for ROs composed of several buffers and one inverter, we calculate the propagation time of a rising and a falling edge of the RO open loop:

(2) $$\begin{array}{c} \tau_{fall}=\alpha_{RO}T_{RO} \end{array}$$

(3) $$\begin{array}{c} \tau_{rise}=\left(1-\alpha_{RO}\right)T_{RO} \end{array}$$

### 2.2. Test Bench Architecture

**Figure 2:**
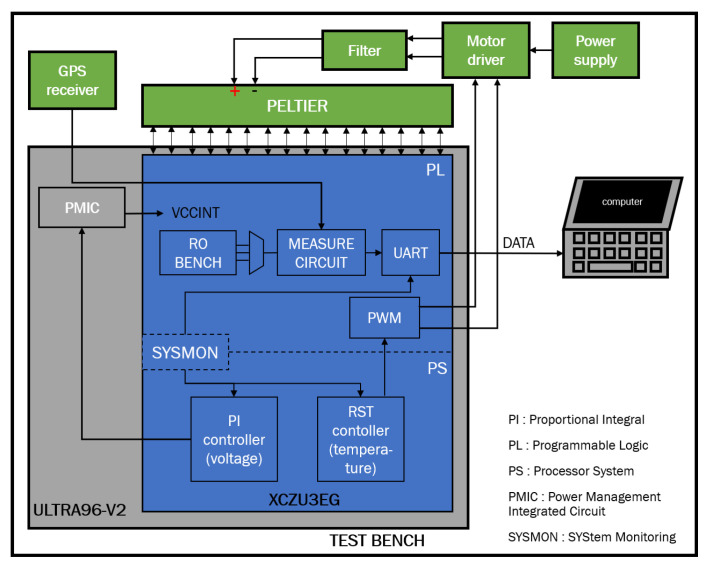
Overview of the FPGA architecture developed in [28].

Figure 2 presents the general architecture of the test bench we used. To measure the effect of 16 nm FinFET degradation in a digital circuit, we used a Zynq UltraScale+ FPGA (blue) designed with FinFET [8]. This FPGA is divided into three parts: the Programmable Logic (PL), the processor system and the SYSMON. SYSMON is a system that measures the FPGA’s internal temperature and voltage. We bought the FPGA already installed on the Ultra96 (grey) development board from Avnet. We developed a regulation system that controls the internal temperature of the FPGA to be between 15 °C and 115 °C with an accuracy of 0.05 °C. We developed another regulation system that controls the internal voltage of the FPGA between $V_{nom}$ (850 mV) and 1.3 $V_{nom}$ with a precision of 3.9 mV. Because the SYSMON’s temperature and voltage measurement is more accurate than regulation, the data are also saved so as to apply postmeasurement compensations. We use a GPS receiver as a time reference in the measurement circuit, as it is more stable than the quartz oscillator generally used. Finally, we showed in [28] that this test bench is able to measure propagation time drifts with a relative accuracy of $9\times 10^{-5}$ . 

### 2.3. Test Strategy

Before beginning ageing, we set the stress conditions: temperature, voltage, stress frequency, stress duty cycle and RO configuration. The choice of appropriate conditions will stimulate specific failure mechanisms.

**Figure 3:**
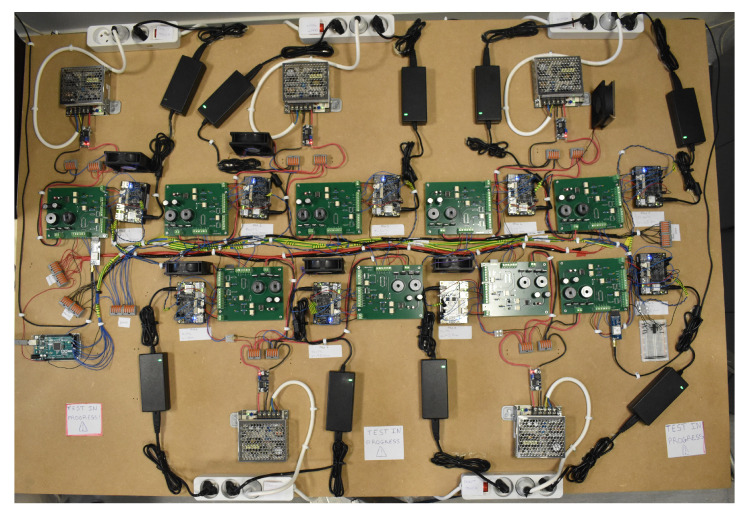
Picture of the test bench for ageing and measuring the degradation of nine FPGAs.

We chose nine FPGAs to be aged, each under different temperature and voltage conditions (see Table 1) so as to model temperature and voltage effect and separate failure mechanism. We selected stress temperatures and voltages close to operational conditions so that the degradation observed is representative of normal use. The internal architecture of each FPGA is identical and corresponds to that shown in Figure 2. A picture of the final test bench is shown in Figure 3.

Thanks to the multiplexer at the input of the RO, we can control the stress signal. To study the effect of internal stress on RO degradation, we selected 21 internal stresses that could be static (DC1, DC0) or dynamic (frequency: 100 Hz, 1 MHz, 100 MHz, 600 MHz and 1500 MHz; duty cycle: 0.1, 0.25, 0.5, 0.75 and 0.9). Static stress will stimulate the BTI, while high-frequency stress will stimulate the HCI. The duty cycle will allow us to study the effect of the BTI recovery mechanism.

In an FPGA, the stages of an RO are implemented by LUTs and the connection between each stage is by the FPGA interconnection network. The LUT is the basic combinational circuit in an FPGA that can implement any combinational logic function by changing the configuration bits. To study the degradation of ROs according to different configurations, we implemented several RO architectures. In order to distinguish degradations in logical (LUT) and routing resources, we implemented two categories of RO: Short Path (SP) and Long Path (LP). The LP consists of two LUTs placed at the opposite sides of the FPGA in order to maximise routing. The SP consists of 13 LUTs implemented in the same logical cell so as to minimise routing. We designed eight different RO SP architectures in order to study the ageing of the LUT as a function of the following:

- The logical function of the LUT: inverter (L1 I), buffer (L1 B), XOR inverter (L2 XI) and XOR buffer (L2 XB);
- The number of LUT inputs used: 3 inputs (L3 XB) and 5 inputs (L5 XB);
- Which input of the LUT is used: input I1 (L3 XB I1) and input I5 (L3 XB I5).

In each FPGA, we have a total of 3ROs×9architectures×21stress=567ROs .

## 3. Results Observation

### 3.1. Measures before Ageing

Before ageing the nine FPGAs, we measured all the RO in operating conditions so as to perform the following:

- Study the RO period distribution in order to extract information on our RO bench;
- Compare with performances expected by the design software Vivado;
- Validate the ability of our test bench to measure RO if the Vivado and measurement results are consistent.

**Figure 4:**
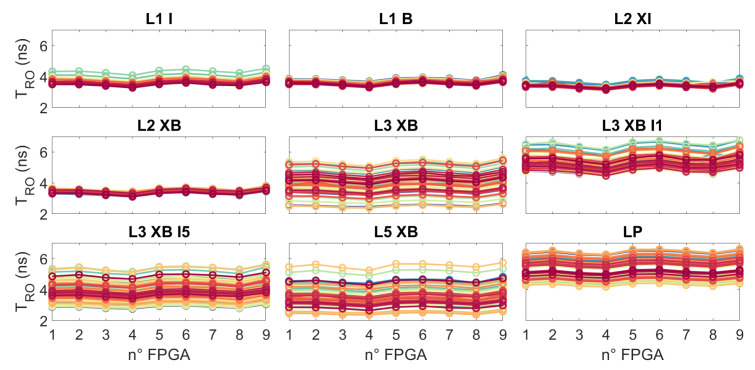
Periods of different RO architectures measured at 25 °C and $V_{nom}$ for 9 FPGAs. Each graphic corresponds to an RO logical architecture (same VHDL code). Each coloured line corresponds to an RO physical architecture (same resources in FPGA).

Figure 4 shows the RO period initial measures. The period of all ROs is between 2 ns and 7 ns, with a frequency between 500 MHz and 150 MHz. Dispersion between RO with the same physical architecture in different FPGAs is less than 10% , which represents inter-FPGA dispersion. Ring oscillators L1 I, L1 B, L2 XI and L2 XB are implemented in one SLICE, while ROs L3 XB, L3 XB I1, L3 XB I5 and L5 XB are implemented in two SLICEs. We can see in Figure 4 that the dispersion is higher for the ROs implemented on 2 SLICEs. This can be explained by more widely available resource choices to ensure the RO routing split on 2 SLICEs has a direct impact on the RO period. We can see that LP ROs have a period close to SP RO (L1 I, L1 B…), which shows the importance of routing for the propagation time of a signal in a path.

**Figure 5:**
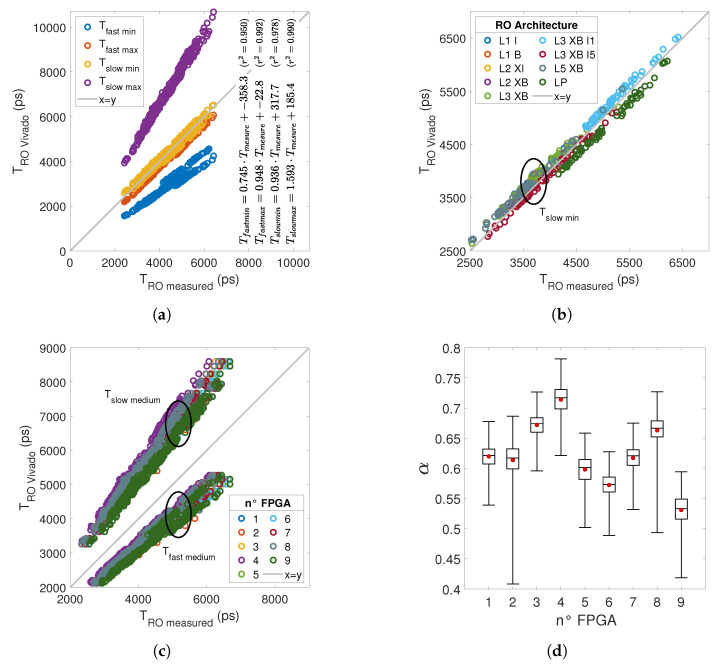
Comparison of the measured and extracted RO period from Vivado for (**a**) all ROs of 1 FPGA, (**b**) Slow Min with a different colour by RO architecture, (**c**) Fast Medium and Slow Medium with different colours by FPGA, and (**d**) dispersion of the alpha coefficient.

Once the ROs are implemented in the FPGA, it is possible to use a TCL script to extract implementation information from Vivado. For every RO, we extracted time propagation given by Vivado which returns four delay references: fastmax , fastmin , slowmax and slowmin . Fast and slow refer to the inter-FPGA variation (process corner) and max and min refer to the intra-FPGA variation. The fastmax delay corresponds to the maximum delay in the fastest FPGA, and the slowmin delay corresponds to the minimum delay in the slowest FPGA. Figure 5a compares RO periods measured with the four delay references given by Vivado: we can see consistency between the measured and Vivado data. The linear factor indicates that measures are close to the slowmin and fastmax references.

So as to observe the differences between RO architectures, we plot the measures with the slowmin reference in Figure 5b with one colour per architecture. By comparing the architectures, we can see that $T_{RO\;Vivado}$ of the LP architecture is underestimated, as well as the L3 XB I5 architecture, whose delay in the LUT is minimal. The LP and L3 XB I5 architectures have in common that routing is the main contributor to delay. We observe this trend on all nine measured FPGAs.

Because the propagation time reference given by Vivado depends on two parameters ( fast/slow and max/min ), it is ambiguous to determine the performance of the FPGA. For example, in Figure 5a, we can see that measurements are close to the slowmin reference (slowest FPGA considering the fastest internal components) but are also close to fastmax reference (fastest FPGA considering the slowest internal components). Therefore, we created two new references which consider intra-FPGA performance as the medium. Thanks to these two references, we know the performance state of our FPGAs considering intra-FPGA performance as the medium. These references are obtained with

(4) $$T_{X,medium}=\frac{T_{X,max}+T_{X,min}}{2}$$

where *X* can be fast or slow .

Figure 5c $T_{ROVivado}$ shows the fastmedium and Slow Medium references as a function of the ROs measurement with one colour per FPGA. It can be seen graphically that FPGA9’s measurements are the farthest from the fastmedium reference and the closest to the slow medium reference, as opposed to FPGA4. We can already tell that FPGA9 seems to be the lowest-performing and FPGA4 the highest-performing. From the data in Figure 5c, we express the measure as a barycentric coordinate with respect to $T_{fast,medium}$ and $T_{slow,medium}$ points such that

(5) $$T_{Measure}=\alpha \cdot T_{fast,medium}+\left(1-\alpha \right)\cdot T_{slow,medium}$$

From Equation (5), we can extract the α proportionality coefficient for every RO in each FPGA:

(6) $$\alpha =\frac{T_{Measure}-T_{slow,medium}}{T_{fast,medium}-T_{slow,medium}}$$

Figure 5d presents the distribution of the α coefficient for each FPGA in an IQR boxplot. Figure 5d shows that the performances of our FPGAs are between 50% and 70% of the best performance expected by Vivado. In Section 5.5, we compare the RO propagation time extracted from Vivado and the measured one after ageing in order to assess whether, after ageing, the propagation time measured remains below Vivado’s acceptable limit.

### 3.2. Measures after Ageing

**Figure 6:**
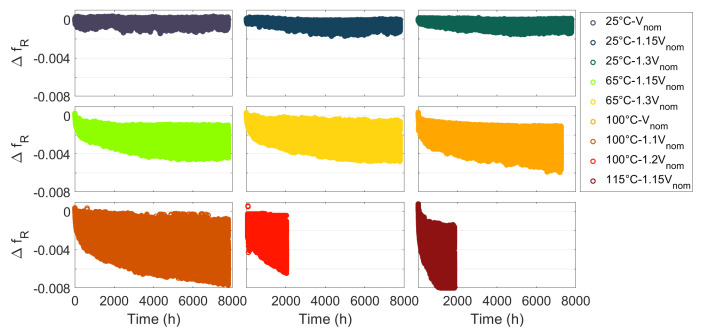
Relative frequency degradation of the 5103 ROs in the nine FPGAs.

After ageing 5103 ROs in nine FPGAs stressed with different temperatures and voltages for 8000 h, Figure 6 presents the results, where

(7) $$\Delta f_{R}\left(t\right)=\frac{f_{RO}\left(t\right)-f_{RO}\left(t_{0}\right)}{f_{RO}\left(t_{0}\right)}$$

We observed that the effect of ageing on the relative frequency of all the ROs is a progressive drift. We did not observe any catalectic failure of the ROs, which suggests that we did not observe a hard TDDB. Figure 6 shows degradations between 0% and 1% . We can clearly see the effect of temperature and voltage on degradations. Thanks to the test bench accuracy, we observe degradation of about 0.2% under stress conditions similar to operating conditions: 25 °C and $V_{nom}$ . Under the same temperature and voltage conditions, we observe dispersed degradation. These differences in degradation demonstrate the significant effect of the FPGA’s internal configuration (stress frequency and duty cycle, RO architecture) on ageing. After approximately 2000 h of the ageing test, the FPGA development boards at 100 °C and 1.2 $V_{nom}$ and 115 °C and 1.15 $V_{nom}$ stopped working, and after around 7500 h of operation, the 100 °C and 1.1 $V_{nom}$ FPGA board stopped working. This is probably due to the failure of a component on the development board.

**Figure 7:**
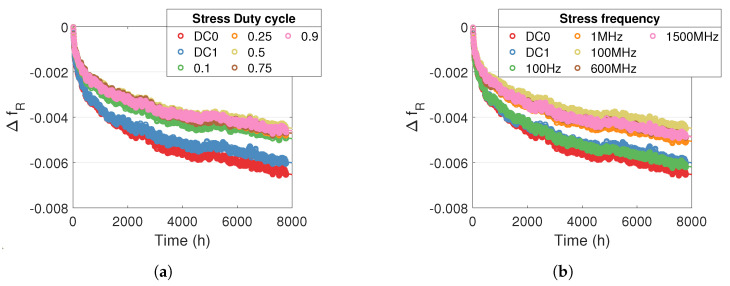
Relative frequency degradation for one RO L1 B with $T_{stress}=100\;{}^{\circ}$ C and $V_{stress}=1.1\;V_{nom}$ (**a**) for different stress duty cycles and stress frequency of 100 MHz (**b**) for different stress frequencies and stress duty cycle of 0.5 .

Figure 7a shows the effect of stress duty cycle on the degradation of the RO relative frequency. We can see that degradation is higher for static stress (DC0 and DC1) than for dynamic stress. The degradations under different stress duty cycles are almost similar, which reveals that the stress duty cycle has no significant effect on the degradation of relative frequency.

Figure 7b shows the effect of stress frequency on the degradation of the RO relative frequency. We observe higher degradations for static stress and for low-frequency stress (100 Hz) than for high-frequency stress where degradations are quite similar for different stress frequencies. The nondependence of degradation on stress frequency means that HCI is not the main failure mechanism causing degradation. In [29] (chapter 21.3.3), the author measures the effect of stress frequency on degradation due to BTI using measurements on isolated transistors. The study shows that the amplitude of the degradations is higher and is dependent on the stress frequency when $f_{stress}\leq 100$ Hz, and the amplitude of the degradations is lower and is independent of the stress frequency when $f_{stress}>100$ Hz. This is exactly what we observed. The author explains that this phenomenon is due to the dynamics of the BTI degradation and recovery mechanism.

Figure 7 shows the degradations for one RO architecture and one temperature and voltage stress; however, we observed the same effect of the duty cycle and stress frequency for the other temperature and voltage conditions. Figure 7 shows higher degradations for DC0 stress than for DC1 stress, which is probably due to the difference between NBTI and PBTI. However, as we do not know the exact internal architecture of the FPGA at the transistor level, we cannot interpret the result any further.

**Figure 8:**
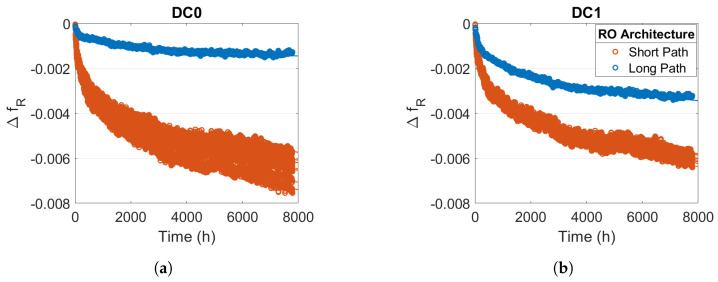
Relative frequency degradation for ROs Long Path (blue) and ROs Short Path (orange) with $T_{stress}=100\;{}^{\circ}$ C and $V_{stress}=1.1\;V_{nom}$ (**a**) for DC0 stress (**b**) for DC1 stress.

An RO SP is composed mainly of logical resources (LUT), whereas an RO LP is composed mainly of routing resources. Figure 8 shows that the degradations are higher for all RO SP than for RO LP, which corresponds to the result obtained in [23]. This observation reveals that in an FPGA, the degradation of logic resources is more sensitive to ageing than the degradation of routing resources. In addition, the degradation of RO LP is more sensitive to DC1 stress than to DC0 stress. Here, again, we can think that this difference is due to NBTI and PBTI.

### 3.3. FPGA Zynq UltraScale+ 16 nm FinFET vs. FPGA Artix 28 nm HKMG

In order to investigate the reliability evolution between an FPGA with planar transistors and an FPGA with FinFET transistors, we compare our measurements and the semiempirical model of the degradations obtained for a Zynq UltraScale+ 16 nm FinFET FPGA with the semiempirical model of the degradations obtained for an Artix 28 nm HKMG FPGA [26]. To predict degradations of the relative frequency of an RO, in [26], the author proposes the following semiempirical model:

(8) $$\Delta f_{R}\left(t,V,T\right)=a\left(V,T\right)t^{b}=A\cdot e^{\gamma \cdot V}e^{-\frac{Ea}{kB\cdot T}}\cdot t^{b}$$

**Figure 9:**
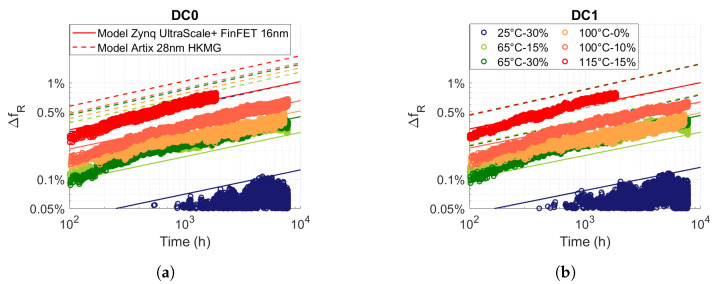
Comparison of the frequency drift degradation of ROs measured (circle) and modelled (full line) for Zynq UltraScale+ 16 nm FinFET FPGAs and modelled (dashed line) for Artix 28 nm HKMG FPGAs for the same temperature and voltage conditions for (**a**) DC0 and (**b**) DC1 internal stress.

Figure 9 shows our measurements (circle) of relative frequency degradation for different temperatures and voltages and for static stress DC0 and DC1. We performed a nonlinear regression between the measurements shown in the figure and the model (8). Figure 9 confirms the consistency between the modelling (full line) and our measurements. Finally, in the figure, we plotted the degradation model obtained in [26] (dashed line), which we adjusted to the same temperature and voltage conditions as our measurements. In general, Figure 9 reveals higher degradation on the Artix 28 nm HKMG than on the UltraScale+ 16nm FinFET for static stress, which is the most stressful condition.

In Table 2, we grouped together the parameters of our model and those obtained in [26]. Firstly, we can see that the time exponent (*b*) is very similar ( 0.24≈0.265 ), which suggests that the failure mechanism is the same. Because the degradations are observed for static stress, for relatively high temperatures and because the time exponent is close to b≈0.25 , like the one characteristic of the BTI reaction–diffusion model, we suspect that BTI is observed in both FPGAs. The acceleration parameters in temperature ( Ea ) and in voltage ( γ ) are different, maybe because of different ageing conditions (temperature, voltage) and modelling methods. But it could also be the consequence of the different physical structures of FinFET and HKMG MOSFET.

The last two lines in Table 2 show the result of the prefactor to time ( a(V,T) ), taking into account the temperature and voltage acceleration factors. We observe a higher amplitude parameter in the Artix model than the one in Zynq UltraScale+, which explains why we observe higher degradations for Artix in Figure 9. Because the degradation in Zynq UltraScale+ is more sensitive to temperature, we can see that for $V=V_{nom}$ and T=25 °C , degradation is ten times lower than in Artix. For $V=1.2\;V_{nom}$ and T=115 °C , the degradations in Zynq UltraScale+ are 1.5 times lower than in Artix.

## 4. A Method for Extracting Degradation in Logical and Routing Resources

### 4.1. Extraction of Logical and Routing Resources

**Figure 10:**
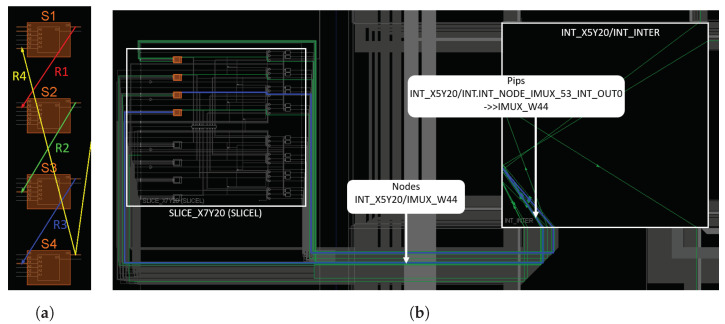
Implementation in the FPGA of the RO shown in Figure 1: (**a**) Simplified floorplan with the LUTs in orange. (**b**) Detailed floorplan with the R3 signal in blue connecting stage S3 to S4.

**Figure 11:**
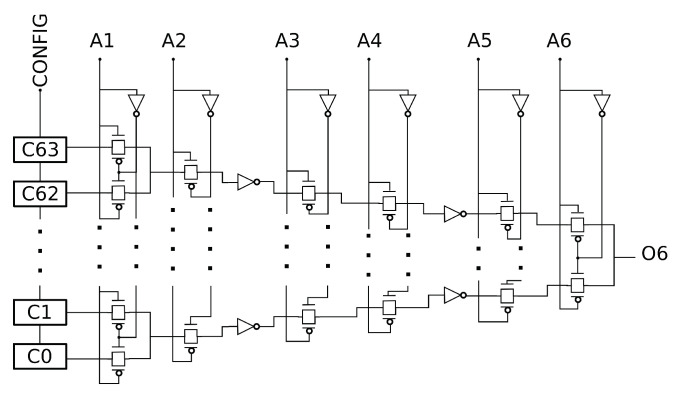
Simplified diagram of the internal architecture of a 6-input LUT based on the patents [30,31].

For the same logical architecture of RO, such as the architecture in Figure 1, Vivado can use different implementations. It can use different LUT inputs or different routing interconnections. Figure 10a represents the simplified floor plan, without the interconnection details, of the FPGA when the four-stage RO of Figure 1 is implemented. Each stage of the RO is implemented by a LUT (in orange), and the signal is routed through one of the six LUT inputs. For example, signal R4 (yellow) uses input A1, while signal R1 (red) uses input A5. Depending on the input used, the signal path through the LUT is more or less lengthy (Figure 11). According to the Xilinx patents [30,31], input A1 corresponds to the longest path and input A6 to the shortest.

Figure 10b shows the detailed floor plan with interconnection details of the FPGA. The four LUTs of the RO are coloured orange. Signal R3 (blue) connects stage S3 to stage S4. To achieve this, the FPGA uses a routing network built around an interconnection matrix. To connect S3 to S4, signal R3 uses nodes and PIPs (Programmable Interconnect Points). A PIP is a configurable circuit that connects two nodes together [32]. For example, PIPs “INT_NODE_IMUX_53_INT_OUT0 −>> IMUX_W44” connects both nodes INT_NODE_IMUX_53_INT_OUT0 and IMUX_W44.

In the FPGA, an RO is implemented by logic resources, which are LUTs with different potential inputs and routing resources consisting of PIPs and nodes. So as to extract from Vivado the resources used by the FPGA to implement each RO, we wrote a TCL script [33].

**Figure 12:**
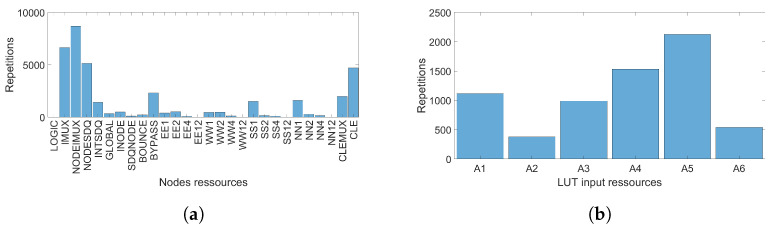
Number of resources used to implement the 567 ROs in one FPGA, classified by category of (**a**) node (**b**) LUT inputs.

Figure 12a displays the number of nodes, by category, used to implement all the ROs. A total of 37,869 node resources are used to implement the 567 ROs. There are a total of 28 node categories, but in our implementation, only 23 node categories are used. The name of the node indicates its direction and length in the routing network. Nodes EE, WW, SS and NN are connections, respectively pointing east, west, south or north in the FPGA. The signal length can be 1 site, 2 sites, 4 sites or 12 sites. The other node categories are local connections between neighbouring logical and interconnection sites. Because a PIP connects two nodes, there are $28^{2}$ PIP categories, which is why we have not plotted the PIP numbers used.

Figure 12b gives the number of LUT inputs per category used to implement all the ROs. A total of 6678 LUT inputs are used. Indeed, we implemented 504 RO SP (Short Path) composed of 13 LUTs and 63 RO LP (Long Path) composed of 2 LUTs.

### 4.2. Initial Propagation Time Extraction

In order to extract the initial propagation times in the logical and routing resources, we performed a regression based on the RO propagation time measurements and the number of resources of each category used in each RO. We assume that the propagation time of a signal in an RO ( $T_{i}\left(t_{0}\right)$ ) corresponds to the sum of the propagation times ( $D_{j}\left(t_{0}\right)$ ) in each of the routing and logic resources that compose the RO ( $R_{i}j$ ):

(9) $$\left[\begin{array}{cccc} R_{11} & R_{12} & \cdots & R_{1m}\\ \vdots & \vdots & \ddots & \vdots \\ R_{n1} & R_{n2} & \cdots & R_{nm} \end{array}\right]\cdot \left[\begin{array}{c} D_{1}\left(t_{0}\right)\\ D_{2}\left(t_{0}\right)\\ \vdots \\ D_{m}\left(t_{0}\right) \end{array}\right]=\left[\begin{array}{c} T_{1}\left(t_{0}\right)\\ T_{2}\left(t_{0}\right)\\ \vdots \\ T_{n}\left(t_{0}\right) \end{array}\right]$$

where $T_{i}$ contains the *i*th RO propagation time given by Vivado (regression 1) or measured (regression 2 and 3), $D_{j}$ contains the propagation time extracted for the *j*th resource category and $R_{ij}$ contains the number of resources of the *j*th resource category used to implement the *i*th RO. The *n* index is the total number of RO (i.e., 567 for regressions 1 and 2 and 5103 for regression 3), and the *m* index is the total number of resource categories. We used the standard ordinary least squares solution $\left(D={\left(R^{t}R\right)}^{-1}R^{t}T\right)$ to obtain the matrix *D* of the system (9) by minimising the residuals $\left(T-R\cdot D\right)$ . If the *D* matrix has negative values, then these same values are set to zero and a new regression is performed. We note that the *R* matrix contains the routing resources (*nodes*) and the logical resources (LUT input). In order to reduce the unknowns in the *R* matrix, we did not use the $28^{2}$ different categories of *PIPs*, since this simplification was proposed by [34]. Therefore, the *m* index is 29 categories, consisting of 23 *node* categories and 6 LUT input categories, as shown in Figure 12.

We performed three linear regressions:

- Regression 1: With the propagation time slowmin of the 567 ROs given by Vivado (see Figure 5a);
- Regression 2: With the propagation time of the 567 ROs measured in one FPGA at T=25 °C and $V=V_{nom}$ ;
- Regression 3: With the initial propagation time of the 5103 ROs measured in nine FPGAs at T=25 °C and $V=V_{nom}$ .

**Figure 13:**
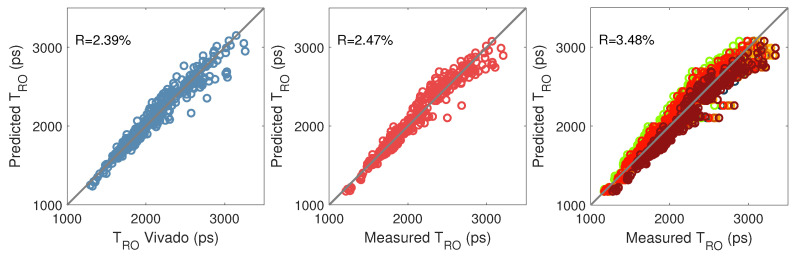
Validation of regressions 1 (**left**) 2 (**middle**) 3 (**right**) expressing R·D as a function of the RO propagation time. In the graph on the right, each colour corresponds to an FPGA.

To confirm the accuracy of the linear regression, Figure 13 compares the predicted propagation time in the ROs ( R·D ) with the propagation time (T) of the ROs given by Vivado (left) or measured in one FPGA (middle) or nine FPGAs (right). We obtained a low relative residual of 2.5% for all the ROs in a single FPGA. The minor noise in the prediction is probably due to two main factors: simplification when categorizing resources (*PIPs* are not included in the resource matrix) and measurement noise. We obtained a slightly larger residual for the prediction of the nine FPGAs because of inter-FPGA variation.

**Figure 14:**
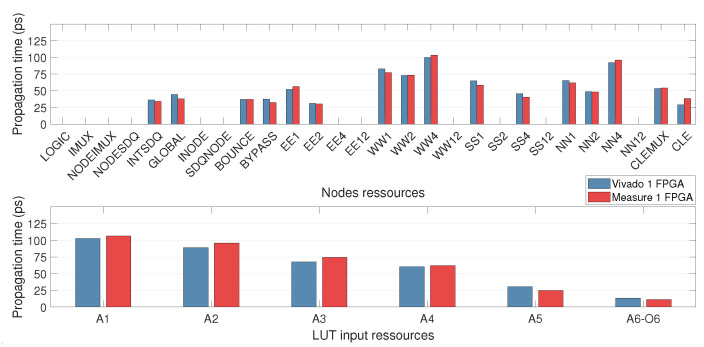
Propagation times of routing resources (**top**) and LUT input resources (**bottom**) corresponding to the *D* matrix, obtained by solving the (9) system with Vivado data (blue) and measurements performed on 1 FPGA (red).

Figure 14 displays the results contained in the matrix *D*, which is obtained by solving the system (9). The results are consistent between the regression based on the Vivado data and the measurements, which confirms the consistency of the extraction method.

Some categories of routing resources have a null result because they are not used or because the resources used are not optimised to solve the system (9).

The routing resources “CLEMUX” and “CLE” are *nodes* that connect the output of the LUT to the input of the interconnection *site*. However, the “CLEMUX” resource passes through an additional multiplexer in the logical site compared with the “CLE”. It can be seen that the propagation time obtained for “CLEMUX” is higher than that for the “CLE” resource, which confirms the consistency of the extraction method.

The LUT resource propagation time results are very consistent with the internal architecture of the LUT (see Figure 11). The longer the path in the LUT, the higher the propagation time (see Table 3). The difference between two adjacent inputs gives the propagation time in one stage of the LUT (see Table 3). We notice that the propagation times of stage 2 and stage 4 are higher than the propagation times of stage 1, stage 3, stage 5 and stage 6. This indicates the potential presence of an inverter between stages 2 and 3 and between stages 4 and 5 in the LUT, as shown in Figure 11. This result is consistent with the patent of the LUT’s internal architecture and confirms our assumption of the LUT’s internal architecture in our FPGA.

### 4.3. Extraction of Propagation Time Degradation

We have shown how to extract, from the period of ROs, the initial propagation time caused by logical and routing resources. The method is now extended by iterating the extraction at each ageing time in order to observe the evolution of the propagation time in both kinds of resources.

In order to reduce the unknowns in the system (9), because routing is composed of wires (nodes) and static transistors (PIPs), we consider that the effect of ageing in the different routing resources is proportional to the propagation time in the initial routing resources. So, we replace in the $R_{m}atrix$ the 23 parameters corresponding to the routing nodes by the propagation time in the initial routing resources.

**Figure 15:**
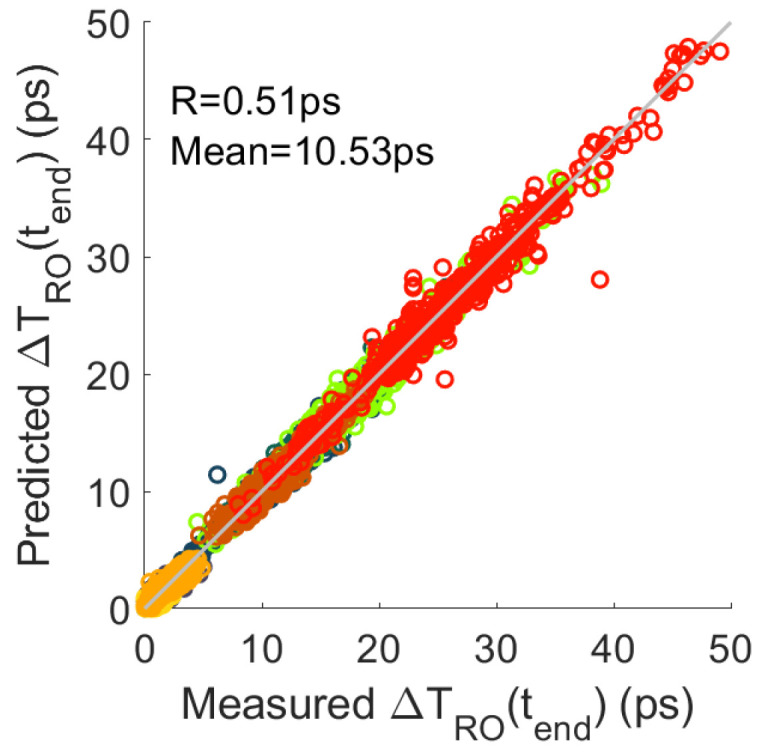
Comparison of predicted and measured RO period degradation at $t_{end}$ in the different FPGAs (colours).

After extracting the propagation time in the logical and routing resources at each measurement moment, Figure 15 shows the comparison of the final degradation ( $\Delta T_{RO}\left(t_{end}\right)=T_{RO}\left(t_{end}\right)-T_{RO}\left(t_{0}\right)$ ) of the measured and predicted RO period. The relative residual of the prediction is 5% , which confirms the accuracy of the degradation extraction method.

**Figure 16:**
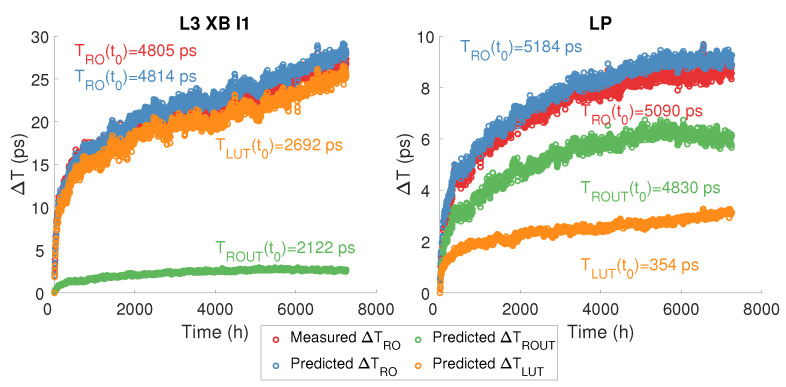
Comparison of measured and predicted L3 XB I1 and LP RO period degradation and decomposition of RO degradation into routing and LUT degradation extracted from prediction.

To confirm the consistency of the method, Figure 16 presents the measured and predicted degradation of the period of two ROs (L3 XB I1 and LP). For RO L3 XB I1, even if the imposed input of the LUT corresponds to the longest logical path, we can see that the initial propagation time generated by the logical ( $T_{LUT}\left(t_{0}\right)$ ) and routing ( $T_{ROUT}\left(t_{0}\right)$ ) resources is almost identical; however, the amplitude of the degradations is about five times greater in the logical resources. For RO LP, because the logical part of the RO is composed of only two LUTs, the initial propagation time generated by the routing resources is 15 times higher than that generated by the logical resources. However, the degradation of routing resources is only three times higher than that of logical resources. Thus, this figure clearly shows that the relative degradations in logical resources are higher than in routing resources, which confirms our observation in Figure 8, which compares the relative degradation of Long-Path and Short-Path ROs.

## 5. Modelling Degradation

In this section, we present and discuss the degradation of rising and falling edge propagation times in an RO under static stress. Then, we model the degradation of the propagation time of the edge in an RO for static and dynamic stress based on the modelling of degradations in logical and routing resources by using data from the extraction method. Finally, we compare the measured degradations with the critical limit set by Vivado to quantify the criticality of the degradations.

### 5.1. Observation and Interpretation

For the circuit timing analysis, the propagation time to be considered is the higher of the two ( $max(\tau_{fall},\tau_{rise})$ ). Because the oscillation period of an RO only gives the average $\frac{tau_{fall}+\tau_{rise}}{2}$ , it tends to underestimate the apparent value of the degradation, especially in the case where one of the two propagation times improves, as we show with static stress. This is why we model the degradation of falling and rising edge propagation times separately.

**Figure 17:**
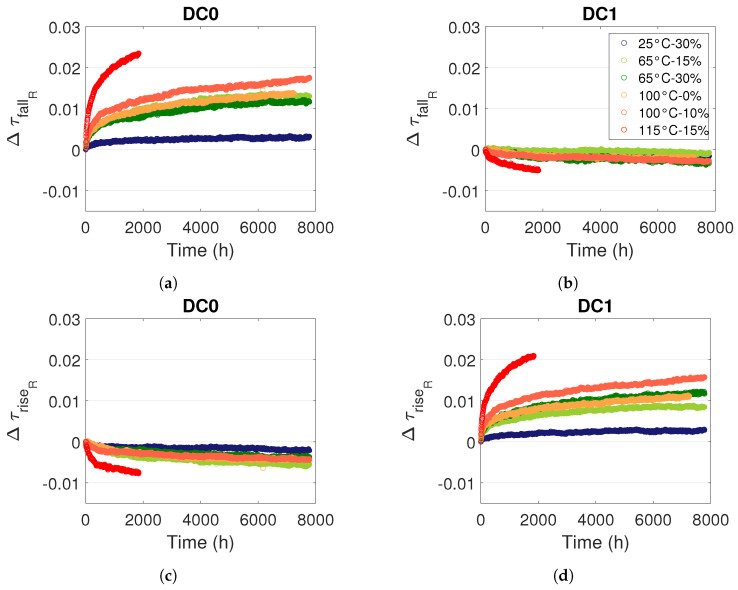
Relative evolution of (**a**,**b**) $\Delta \tau_{fall_{R}}$ and (**c**,**d**) $\Delta \tau_{rise_{R}}$ in an RO L1 B for different temperature and voltage stress and internal stress signals (**a**,**c**) DC0 and (**b**,**d**) DC1.

Figure 17 shows the degradation of rising and falling edge propagation times in the RO under static stress DC0 and DC1 for different voltage and temperature conditions. In Figure 17a (DC0, $\tau_{fall}$ ) and Figure 17d (DC1, $\tau_{rise}$ ), we observe a propagation time increase (degradation), while in Figure 17b (DC1, $\tau_{fall}$ ) and Figure 17c (DC0, $\tau_{rise}$ ), we observe a propagation time decrease (improvement). This behaviour can be explained by considering the stress of each transistor and its different effect on $\tau_{rise}$ and $\tau_{fall}$ . Indeed, an RO is composed of LUTs and routing resources that use transmission gates and inverters (see Figure 11). When the passing transistors for static stress are the same as the passing transistors for transmitting the measured signal, for example, a DC0 stress and the $\tau_{fall}$ measurement, then the propagation time in an inverter and a transfer gate increases, and therefore, the propagation time of the measured edge in the RO increases (see Figure 17a,d). When the passing transistors for static stress are different to the passing transistors for transmitting the measured signal, for example, a DC0 stress and the $\tau_{rise}$ measurement, then the propagation time in an inverter decreases, and the propagation time in a transfer gate does not change significantly; thus, the propagation time of the measured edge in the RO decreases (see Figure 17b,c). More detailed explanations are given in [35].

While Figure 6 shows relative degradations of RO frequency lower than 1% , Figure 17 shows that relative degradations of edge propagation time can be higher than 2% , which confirms the importance to dissociate rising and falling edge propagation time.

### 5.2. Modelling Logical Resources Degradations under Static Stress

**Figure 18:**
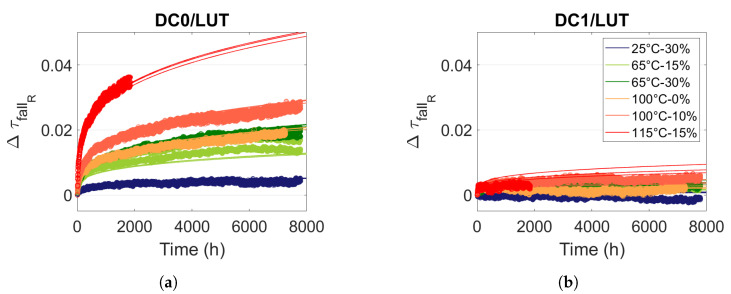
Measure and model (12) of relative evolution $\Delta \tau_{fall_{R}}$ in LUT resources for different temperature and voltage stress and internal stress signals (**a**) DC0 and (**b**) DC1.

So as to predict propagation time degradations in logical resources, we first model them under static stress. Figure 18 displays the evolution of the propagation time of a falling edge in the LUT resources for a static stress DC0 and DC1. Figure 18a shows that the relative degradations are higher in the LUT resources than in the RO (see Figure 17a), which further confirms that the relative degradations in the logical resources are higher than in the routing resources. The propagation time improvement in the LUT resources (see Figure 18b) is smaller than in the RO (see Figure 17b). Propagation time improvements are only produced in an inverter. However, in a LUT, the number of inverters is low compared with the number of transmission gates. This explains the small improvements in the propagation time (less than 0.1% ).

BTI models of threshold voltage degradation under static stress are well-established [36,37]. Because the propagation time of a signal in a transistor is proportional to its threshold voltage, we use the following model with Arrhenius law for temperature acceleration factor and exponential law for voltage acceleration factor [26]:

(10) $$\Delta \tau_{R}=Ge^{\gamma \left(V_{stress}-V_{op}\right)}e^{\frac{Ea}{k_{B}}\left(\frac{1}{T_{op}}-\frac{1}{T_{stress}}\right)}t^{b}$$

where *G* is the amplitude factor, γ is the electrical acceleration parameter, $V_{op}$ is the nominal voltage of 0.85 V, $V_{stress}$ is the stress voltage, Ea is the energy of activation, $k_{B}$ is Boltzmann’s constant, $T_{op}$ is the nominal temperature of 298 K and $T_{stress}$ is the stress temperature. We note in Table 4 the extracted parameters γ , Ea and *b*, which are similar for all RO architectures.

The amplitude factor (*G*) is different for each LUT. However, we observed that the amplitude factor *G* is not only relative to the initial propagation time in the LUT but also to the LUT input used. Depending on the input used (as shown in Section 4), the signal passes through different numbers of PMOS and NMOS transistors, which can have different failure mechanisms. We are looking for the amplitude factor specific to each LUT input such that

(11) $$\left[\begin{array}{ccc} R_{ILUT_{A1,1}} & \cdots & R_{ILUT_{Am,1}}\\ \vdots & \cdots & \vdots \\ R_{ILUT_{A1,n}} & \cdots & R_{ILUT_{Am,n}} \end{array}\right]\cdot \left[\begin{array}{c} C_{ILUT_{A1}}\\ \vdots \\ C_{ILUT_{Am}} \end{array}\right]=\left[\begin{array}{c} G_{1}\cdot \tau_{t_{0}1}\\ \vdots \\ G_{n}\cdot \tau_{t_{0}n} \end{array}\right]$$

where $R_{ILUT_{A1,n}}$ is the number of repetitions of the LUT input Am in the *n*th RO, $C_{ILUT_{A1}}$ is the absolute amplitude factor of the LUT input, $G_{n}$ is the relative degradation amplitude factor of LUT of the *n*th RO and $\tau_{t_{0}n}$ is the initial propagation time in the LUTs of the *n*th RO. By applying a linear regression using the standard least squares method, we obtain the vector $C_{ILUT}$ . We use the absolute amplitude degradation factor ( $G_{n}\times \tau_{t_{0}n}$ ) to base the regression on a physical relationship.

Finally, we obtained four models to predict the evolution of the edge propagation time *F* (fall or rise) for a static stress *S* (DC0 or DC1) in the logic resources ( lut ) for all RO architectures:

(12) $$\Delta \tau_{R_{F,S,lut}}\left(t\right)=\frac{R_{ILUT}\times C_{ILUT}}{\tau_{LUT}\left(t_{0}\right)}\times e^{\gamma \left(V_{stress}-V_{op}\right)}e^{\frac{Ea}{k_{B}}\left(\frac{1}{T_{op}}-\frac{1}{T_{stress}}\right)}t^{b}$$

Figure 18 illustrates the consistency of the model (solid line) with the degradation measurements in the logical resources.

### 5.3. Modelling Routing Resources Degradations under Static Stress

**Figure 19:**
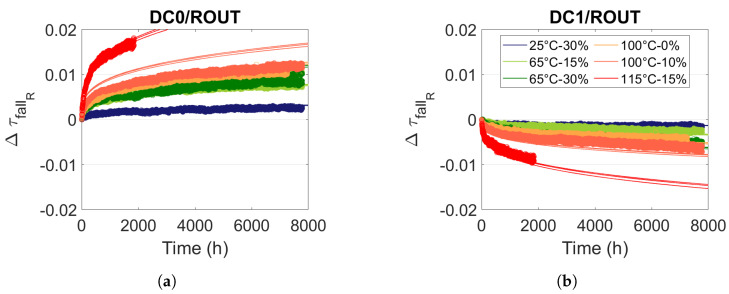
Measure and model (14) of relative degradation $\Delta \tau_{fall_{R}}$ in routing resources for different temperature and voltage stress and internal stress signals (**a**) DC0 and (**b**) DC1.

So as to predict propagation time degradation in routing resources, we first model them under static stress. Figure 19 shows propagation time degradations in routing resources. Comparison with Figure 18 reveals that degradations are two times lower in routing resources than in logical resources. However, improvements are significantly higher in routing resources. Considering that the effects of propagation time improvements are present in inverters, the last observation suggests that in routing resources, the ratio between the number of inverters and transfer gates is greater than in LUT resources.

We modelled degradations and improvements in routing resources with the model (10). We note in Table 4 the parameters γ , Ea and *b*, which are similar for all RO architectures. However, we observed that the amplitude coefficient *G*, which is relative to the initial propagation time, increases as the propagation time decreases. Note that a routing resource is composed of wire and PIPs, which are made up of transistors. Since a routing resource needs a minimum number of PIPs to be connected, the propagation time generated by the PIPs ( $\frac{\tau_{pips}}{\tau_{pips}+\tau_{wires}}$ ) is proportionally higher for a Short Path than for a Long Path. However, degradation is generated by failure mechanisms in the transistors of PIPs. Consequently, it is consistent that the amplitude of the degradations relative to the initial propagation time is higher when the initial propagation time is lower. We have modelled this dependency with a first-degree polynomial such that

(13) $$G=G_{t_{0}}\times \tau \left(t_{0}\right)+B$$

Hence, we have four models to predict the evolution of the edge propagation time *F* (fall or rise) for a static stress *S* (DC0 or DC1) in the routing resources ( rout ) for all RO architectures:

(14) $$\Delta \tau_{R_{F,S,rout}}\left(t\right)=\left(G_{t0}\tau \left(t_{0}\right)+B\right)\times e^{\gamma \left(V_{stress}-V_{op}\right)}e^{\frac{Ea}{k_{B}}\left(\frac{1}{T_{op}}-\frac{1}{T_{stress}}\right)}t^{b}$$

Figure 19 illustrates the consistency of the model (full line) with the degradation measurements in the logical resources.

### 5.4. Modelling Routing and Logical Resources Degradations under Dynamic Stress

In order to extend the previous static model to dynamic stresses, we first attempt to model ageing directly from the static model. Because the method for modelling degradations in logical and routing resources for dynamic stress is the same, we only show the results for routing resources. Remember that $\tau_{R_{F,S,rout}}\left(t\right)$ (14) predicts the evolution of the edge propagation time *F* (rise or fall) for a static stress *S* (DC0 or DC1) in the routing resources. We consider that the degradation with dynamic stress corresponds to the sum of the degradations obtained with the static stresses DC0 and DC1 as a function of the time spent by the stress signal at the high and low levels, and therefore as a function of the duty cycle of the dynamic stress signal. Thus, the $Model_{R_{F,rout}}$ predicts the degradation of the edge propagation time *F* in the routing resources:

(15) $$\Delta \tau_{R_{F,rout}}\left(t,\alpha_{stress}\right)=\Delta \tau_{R_{F,DC1,rout}}\left(\alpha_{stress}t\right)+\Delta \tau_{R_{F,DC0,rout}}\left(\left(1-\alpha_{stress}\right)t\right)$$

The term $(1-\alpha_{stress})t$ is the ageing time with stress DC0, and $\alpha_{stress}t$ is the ageing time with stress DC1.

**Figure 20:**
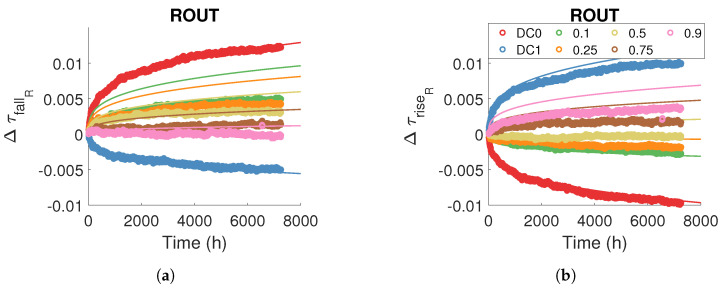
Measure and model (15) of relative degradation (**a**) $\Delta \tau_{fall_{R}}$ (**b**) $\Delta \tau_{rise_{R}}$ in routing resources for a dynamic internal stress signal with a frequency of 100 MHz and different stress duty cycles. $T_{stress}=100\;{}^{\circ}C$ and $V_{stress}=V_{nom}$ .

Figure 20 compares the model defined in Equation (15) with the measurements. For static stresses DC0 and DC1, the static model defined in the previous section directly predicts the ageing effect. For dynamic stresses, the model overestimates degradation. The difference between the measurements and the model is probably due to the BTI recovery effect, which we did not include in this first model. Both in routing and LUT resources, depending on the logic level to be transmitted, some transistors are passed and others are blocked, so some transistors are degraded and others recovered. In the case of dynamic stress, the degradation we measure is actually the sum of two phenomena: degradation and recovery due to the BTI.

We now model the recovery effect to accurately predict degradation under dynamic stress. The BTI recovery effect for high- κ and trigate technologies is studied in [38,39]. In these studies, the authors propose an empirical model in which the recovery effect is modelled as a Recovery Fraction (RF):

(16) $$RF=\frac{1}{1+B{\left(\frac{t_{recovery}}{t_{stress}}\right)}^{\beta }}$$

where *B* and β are fitting data. In our case, for stress level ‘0’: $t_{recovery}=t_{high}$ and $t_{stress}=t_{low}$ , whereas, for stress level ‘1’, $t_{recovery}=t_{low}$ and $t_{stress}=t_{high}$ . Considering that $\alpha_{stress}=\frac{t_{high}}{t_{high}+t_{low}}$ :

(17) $$RF_{0}=\frac{1}{1+B_{0}{\left(\frac{\alpha_{Stress}}{\left(1-\alpha_{stress}\right)}\right)}^{\beta_{0}}}$$

(18) $$RF_{1}=\frac{1}{1+B_{1}{\left(\frac{\left(1-\alpha_{stress}\right)}{\alpha_{Stress}}\right)}^{\beta_{1}}}$$

Thus, the following model predicts the degradation of the edge propagation time *F* in the routing resources taking into account the recovery:

(19) $$\Delta \tau_{R_{F,rout}}\left(t,\alpha_{stress}\right)=\Delta \tau_{R_{F,DC1,rout}}\left(\alpha_{stress}t\right)\cdot RF_{1}+\Delta \tau_{R_{F,DC0,rout}}\left(\left(1-\alpha_{stress}\right)t\right)\cdot RF_{0}$$

We applied a nonlinear regression for each temperature and voltage condition between the (19) model and degradation measurements for different stress frequencies and duty cycles to identify the $B_{0}$ , $B_{1}$ , $\beta_{0}$ and $\beta_{1}$ parameters common to all RO architectures. We observed a temperature and voltage dependence of the parameters $B_{0}$ , $B_{1}$ , $\beta_{0}$ and $\beta_{1}$ , which we modelled empirically with a two-variable polynomial of second order.

**Figure 21:**
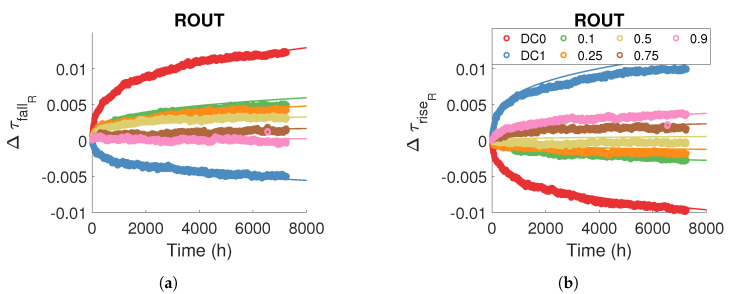
Measure and model (19) of (**a**) $\Delta \tau_{fall}$ (**b**) $\Delta \tau_{rise}$ in routing resources for a dynamic internal stress signal with a frequency of 100 MHz and different stress duty cycles. $T_{stress}=100\;{}^{\circ}C$ and $V_{stress}=V_{nom}$ .

Figure 21 illustrates the consistency of the model (19) for predicting the propagation time in routing resources for static and dynamic stress. We did not observe any effect of stress frequency on degradation amplitude.

Finally, we developed two models to predict the edge propagation time *F* (fall or rise) in logical resources ( $Model_{F,lut}$ ) and in routing resources ( $Model_{F,rout}$ ). By summing these two models, we obtain the model for predicting the edge propagation time in an RO:

(20) $$Model_{F,RO}=Model_{F,lut}+Model_{F,rout}$$

**Figure 22:**
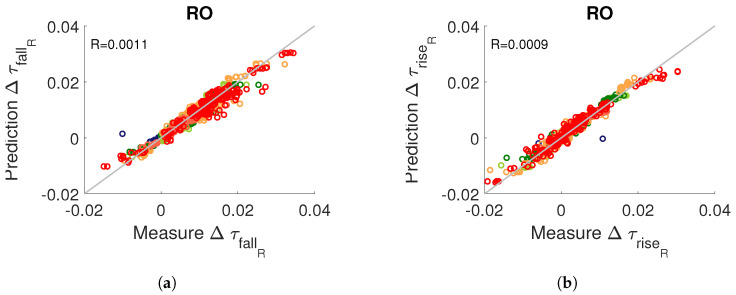
Comparison between measurement and prediction with model (20) of degradations in propagation time, at $t_{end}$ , for (**a**) falling and (**b**) rising edge in ROs for all static, dynamic, temperature and voltage stress conditions (different colours) and for all RO architectures.

Figure 22 compares the evolution of the rising and falling edge propagation time in an RO at the last measurement time with the prediction model (20) for all RO architectures, all static and dynamic stresses and all temperature and voltage conditions. With a relative residual of less than 10% , this figure confirms the accuracy of our prediction model.

### 5.5. Degradation Measured vs. Vivado’s Critical Limit

The maximum degradations we observed on the 5103 ROs are slightly higher than 2% for the degradation of a falling edge in an RO stressed by DC0 and for $T_{stress}=115\;{}^{\circ}C$ and $V_{stress}=1.15\;V_{nom}$ . In this section, we compare the degradations we measured in the worst case (DC0 and $\tau_{fall}$ ) with the critical propagation times set by Vivado. Using the propagation times we extracted from Vivado ( fastmax , fastmin , slowmax and slowmin ), we calculate the maximum propagation time in an FPGA with average performance accepted by Vivado:

(21) $$\tau_{vivado}=\frac{\tau_{fast,max}+\tau_{slow,max}}{2}$$

**Figure 23:**
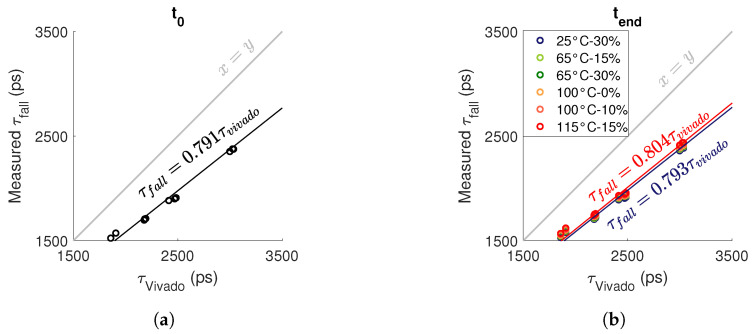
Comparison of the propagation time extracted from Vivado (21) with the measurement of a falling edge (**a**) before ageing and (**b**) after ageing for a DC0 stress. Each point corresponds to an RO.

Figure 23 compares the critical propagation time set by Vivado with the propagation time of a falling edge measured before and after ageing. Before ageing, we can see that Vivado’s critical propagation time is 1.21 times higher than the measured propagation time, which means there is a 21% margin for degradation. Figure 23b clearly demonstrates that after ageing, the maximum degradations we measured (≈2%) are considerably lower than the margin set by Vivado (≈21%). By extrapolating the measurements, we obtain for $T_{stress}=115\;{}^{\circ}C$ and $V_{stress}=1.15\;V_{nom}$ that half of the ROs will reach the limit set by Vivado after $10^{6}$ years. This proves that the reliability of FPGAs under the effect of ageing of transistors is far from being a problem capable of altering the operation of an FPGA.

## 6. Conclusions

To evaluate the reliability of 16nm FinFET digital circuits, the degradations of 5103 ROs distributed over nine FPGAs, each with a different temperature and voltage stress, were aged and measured for 8000 h.

Before ageing, all the ROs were measured and compared with the expectations of the design software (Vivado). From this comparison, the initial performance of the FPGAs between 50% and 70% of the best performance expected by Vivado was revealed. After ageing for 8000 h, oscillation frequency drifts of up to 1% on all the ROs were observed. Higher degradations when the RO was subjected to static stress were measured, which suggests that BTI is dominant over HCI at temperatures of 25 °C or higher. By implementing different RO architectures, higher degradations were observed when the RO was composed mainly of logical resources rather than routing resources. The degradations we measured on a 16 nm FinFET FPGA were compared with those obtained for a 28 nm MOSFET FPGA. With degradations 1.5 times lower when $T_{stress}=115\;{}^{\circ}C$ and $V_{stress}=1.2\;V_{nom}$ and for static stress, a better reliability of the 16 nm FinFET FPGA was revealed by this comparison.

A method was developed to identify, from the propagation time measured on an RO, the propagation time produced by logical and routing resources. By iterating this identification at each measurement instant, propagation time degradations in logical and routing resources with an accuracy of 5% were obtained. Using this method, relative degradations in logical resources around five times higher than degradations in routing resources were reported.

By studying the evolution of the propagation time of a rising and falling edge in an RO, an effect of degradation but also of improvement of the propagation time was observed depending on the internal stress signal used. A model for predicting the evolution of propagation time in an RO as a function of ageing time, temperature, voltage, stress duty cycle and the FPGA resources used was developed and a prediction accuracy of less than 10% was achieved. By comparing our propagation time degradations (maximum 2% ) with the critical degradation limits set by the design software ( 21% ), the negligible effect of ageing on the operation of an FPGA, and more generally of a digital circuit, was highlighted. A mean time of around $10^{6}$ years was obtained by extrapolating the measurements up to 21% degradations in propagation time, which is insignificant.

## Figures and Tables

**Table 1:** FPGA stress conditions.

FPGA	1	2	3	4	5	6	7	8	9
T (°C)	25	25	25	65	65	100	100	100	115
** $\frac{V}{V_{nom}}$ **	1	1.15	1.3	1.15	1.3	1	1.1	1.2	1.15

**Table 2:** Degradation model parameters (8) extracted for a Zynq UltraScale+ 16 nm FinFET FPGA and an Artix 28 nm HKMG FPGA.

	Zynq UltraScale+ 16 nm FinFET		Artix 28 nm HKMG	
	**DC0** ( $R^{2}=0.04$ )	**DC1** ( $R^{2}=0.04$ )	**DC0**	**DC1**
*A* ( $\times 10^{2}\cdot h^{-b}$ )	−21.3	−15.3	−0.592	−0.066
*b*	0.251	0.240	0.262	0.265
Ea (eV)	0.275	0.268	0.089	0.160
γ ( $V^{-1}$ )	2.957	3.156	1.231	4.804
$a(V_{nom}$ , 25 °C)	−5.9	−6.6	−64.0	−15.6
( $\times 10^{3}\cdot h^{-b}$ )				
$a(1.2V_{nom}$ , 115 °C)	−117	−127	−182	−174
( $\times 10^{3}\cdot h^{-b}$ )				

**Table 3:** Propagation time for different LUT inputs extracted by linear regression (9).

A1	104 ps	stage 1: A1–A2	13 ps
A2	91 ps	stage 2: A2–A3	22 ps
A3	69 ps	stage 3: A3–A4	7 ps
A4	62 ps	stage 4: A4–A5	31 ps
A5	31 ps	stage 5: A5–A6	17 ps
A6	14 ps	stage 6: A6	14 ps

**Table 4:** Parameters of the model (10) for the propagation time degradation in LUT and routing resources.

	Degradation			Improvement		
	γ **(V^−1^)**	Ea **(eV)**	b	γ **(V^−1^)**	Ea **(eV)**	b
LUT	4.21	0.31	0.27	5.98	0.31	0.24
ROUT	5.17	0.29	0.29	3.16	0.34	0.29

## Data Availability

Data are contained within the article. For reasons of confidentiality, we cannot provide any further details on the data.

## Abbreviations

The following abbreviations are used in this manuscript:

BTI	Bias Temperature Instability
FPGA	Field-Programmable Gate Array
HCI	Hot Carrier Injection
LP	Long Path
LUT	Look-Up Table
PIP	Programmable Interconnect Point
RO	Ring Oscillator
SHE	Self-Heating Effect
SP	Short Path
TDDB	Time-Dependent Dielectric Breakdown
